# Inter-alpha-trypsin inhibitor heavy chain 4 (ITIH4) in saliva of pigs: evaluation of two commercially available ELISA kits for its measurement and distribution of its main components

**DOI:** 10.1371/journal.pone.0335133

**Published:** 2025-10-24

**Authors:** Alba Ortín-Bustillo, Silvia Martínez-Subiela, José Joaquín Cerón, Antonio González-Bulnes, Asta Tvarijonaviciute, Alberto Muñoz-Prieto

**Affiliations:** 1 Salilab-UMU, Interdisciplinary Laboratory of Clinical Analysis (Interlab), Regional Campus of International Excellence ‘Campus Mare Nostrum’, University of Murcia, Murcia, Spain; 2 Department of Animal Health and Production, Faculty of Veterinary, Universidad Cardenal Herrera-CEU, CEU Universities, C/Tirant lo Blanc, 7, Alfara del Patriarca, Valencia, Spain; Consiglio Nazionale delle Ricerche, ITALY

## Abstract

Inter-alpha-trypsin inhibitor protein heavy chain 4 (ITIH4), also named porcine major acute phase protein (Pig-MAP), is a positive acute phase protein (APP) in pigs and can be measured in plasma and also in saliva to assess the animal health. The objectives of this report were (1) to evaluate if different commercially available porcine ELISA kits can measure ITIH4 concentrations in saliva samples of pigs, and (2) to study the distribution of ITIH4 components in saliva and compare it to the distribution in plasma. The results showed that two of the ELISA kits used in this report could measure salivary ITIH4 with precision and accuracy, but only one showed significant differences between pigs with tail biting and control animals without this condition. Western blotting analysis revealed the presence of a different distribution of ITIH4 bands in saliva samples compared to plasma samples. In conclusion, in saliva of pigs ITIH4 can be quantified using a commercial ELISA kit increasing its concentration in cases of tail biting. In addition, ITIH4 shows bands at western blot in saliva that are different from serum but are compatible with different ITIH4 forms. These data confirm that ITIH4 can be detected in saliva and be potentially used as a biomarker of inflammation.

## Introduction

Inter-alpha-trypsin inhibitor protein heavy chain 4 (ITIH4) is a serum glycoprotein that belongs to the Inter-alpha-trypsin inhibitor protein family (ITI) [1]. In human serum, it was identified by several names, such as a serum glycoprotein 120 kDa (sgp120), and ITI heavy chain human-related protein (IHRP) [2,3]. In addition, as it is a plasma kallikrein-sensitive protein, it was called PK-120 [4,5]. In porcine, ITIH4 is also known as pig IHRP [6] or porcine major acute phase protein (PigMAP) [7]. In both humans [8] and pigs [6], different bands or components of ITIH4 have been described in plasma.

ITIH4is considered an acute phase protein (APP) that increases after any inflammation. In humans, it is used in plasma or serum to detect and monitor inflammation and bacterial systemic infections, such as increased levels in patients who have ongoing bacteraemia [9]. Besides, other researchers have suggested the potential of this biomarker as a tool to diagnose early gastric cancer with a specificity of 94.44% [10]. In swine, a previous work reported significant differences in ITIH4 levels in serum at different ages [11], other study has shown that the levels of ITIH4 in serum increase in animals with disease [12], and also higher levels of ITIH4 in pig serum were observed after long duration transport used as a stress model [13]. This APP also increases in the pig serum after the inoculation of toxigenic strain of *Pasteurella multocida,* [14]. Furthermore, a porcine model has been used to evaluate serum ITIH4 as a marker to monitor hepatocellular carcinoma complications in non-alcoholic fatty liver disease [15].

In addition to plasma or serum, ITIH4 can be analysed in the saliva of pigs, which is a sample that is gaining attention due to its easy collection and where other biomarkers related to stress, immune system and redox status can be measured [16]. ITIH4 quantified by a commercially available kit showed higher levels in saliva in cases of diarrhoea due to *Escherichia coli* (*E.coli*) [17], and meningitis by *Streptococcus suis* (*S.suis*) infection [18], and also under stress situations [19]. Recently, an in-house method has been developed to measure ITIH4 in the saliva of this species [20]. Nevertheless, although ITIH4 has been quantified in saliva, there is no data available about the different forms of this protein in saliva and whether these forms can differ from those found in serum.

The objective of the present study was to provide further knowledge about the presence and possibilities to measure ITIH4 in saliva, and for this purpose, we had two sub-objectives: (1) evaluate whether four different commercially available porcine ELISA kits for serum ITIH4 quantification could be used to measure this analyte in saliva, and (2) to study the distribution of ITIH4 components in saliva and compare it to the distribution in plasma. It is expected that this information will be of use for the measurement and interpretation of this APP in saliva.

## Materials and methods

### Animals

Samples from three different groups of pigs (*Sus Scrofa domesticus*, Landrance x Large-White) were used in this report:

- Samples from pigs located at the Veterinary Teaching Farm of the University of Murcia (Spain) were used to purify the native ITIH4 porcine protein. These pigs were in their growing-finishing phase (100 days, 60 kg mean body weight) and were vaccinated against *Mycoplasma hyopneumoniae* and Porcine circovirus type 2 at weaning, with the farm being free for porcine respiratory and reproductive syndrome virus (PRRSv).
- Samples from pigs from a previous study [17], which were used in this report for the kits analytical validation study and also for western blotting (WB) analysis. From these samples, two pools of saliva samples were used: one made from individual saliva samples from piglets with diarrhoea due to *E. coli* (n = 7) and the second one made from healthy piglets (n = 7) in order to have samples with different concentrations of ITIH4 in saliva. All animals were weaning pigs, and the diseased group showed moderate to severe clinical signs compatible with the diarrheic syndrome. Rectal swabs were used to detect the presence of *E. coli* F4, and positive to heat-labile toxin confirmed by multiplex PCR [21]. The pigs of the healthy group were negative for the *E. coli* F4 and heat-labile toxin. In addition to the saliva samples, serums from these pigs were also used for WB analysis.
- Saliva samples from a previous study [22] were used to evaluate the ability of assays that had been shown at the analytical validation to be capable of quantifying ITIH4 in saliva to detect an inflammatory condition such as tail biting. Animals of the group with inflammation had signs of fresh tail biting (TB, n = 13), whereas pigs from the control group had no tail lesions (C, n = 14) [22]. Pigs were of similar age (8–9 weeks old) and were allocated to a growing unit of a commercial piglet producing farm in Southwest Finland. To confirm the presence of inflammation in these animals and also to compare and study its correlation with ITIH4, the values of salivary porcine haptoglobin (Hp) were used, which is also a moderate APP in pigs [23]. It was measured previously in the animals of this report [22] and the means were 1879.68 ng/mL in the C group, and 8108.15 ng/mL in the TB group. All samples were stored at −80ºC.

The Ethical Committee on Animal Experimentation (CEEA) of the University of Murcia approved the research protocol in this study with approval number CEEA-937/2024, according to the European Council Directives regarding the protection of animals used for experimental purposes. Also, this study complies with ARRIVE guidelines for the care and use of animals. Informed consent was obtained from the farm owners where samples of pigs were collected.

### Saliva and plasma sampling

Saliva samples were collected using saliva collection tubes (Salivette, Sarstedt, Aktiengesellschaft & Co., Nümbrecht, Germany) and synthetic sponges, as previously reported [24]. In brief, each animal was allowed to chew gently a sponge (size 5 cm long; 2.5 cm wide), which was attached to a thin, flexible metal rod, until the sponge was thoroughly moistened around 1–2 min. Each sponge was introduced into a Salivette tube and transported on ice to the laboratory. Once in the laboratory, tubes were centrifuged (3000 g, 10 min, 4 ◦C), and saliva was transferred to 1.5 mL tubes (Eppendorf Ibérica, Spain) and stored at −80 ◦C until analysis. The average time from collecting until centrifugation was around 2 h.

Blood samples were collected by jugular vein-puncture using a vacutainer, heparin tubes (BD Vacutainer, Franklin Lakes, NJ, USA), and needles 25 mm × 0.8 mm (BD Vacutainer, Franklin Lakes, NJ, USA). Plasma was separated by centrifugation at the laboratory (2000 g, 15 min, 4 ◦C), transferred to 1.5 mL tubes (Eppendorf Ibérica, Spain) and stored at −80 ◦C until its use.

### ELISA assays

Four ELISA kits commercially available to analyse ITIH4 in porcine plasma were evaluated to see if there was reactivity using porcine saliva. The kits were selected because they were available in the market for the measurement of ITIH4 in pigs. Details of the kits appear in Table 1. All samples were prepared following the instructions of each kit and were diluted with the sample buffer provided. In case of the kits validated, the dilution was 1/25 and 1/2 for the C and D ELISA kits, respectively.

**Table 1:** Specific commercial ELISA assays used in this report available to measure ITIH4 in serum porcine samples.

ELISA kit	Name	Reference	Type	Range	Sensitivity
A	Fine test Pig MAP	EP0218	Sandwich	0.156-10 ng/mL	0.094 ng/mL
B	Acuvet ELISA pig-MAP	AC/PME01	Sandwich	0.3-3 μg/mL	0.2 μg/mL
C	Cusabio Pig MAP	CSB-E13425p	Competitive	37.5 ng/mL-2400 ng/mL	9.38 ng/mL
D	Elabscience Porcine ITIH4	E-EL-P0304	Sandwich	3.13-200 ng/mL	1.88 ng/mL

**MAP: major acute protein; ITIH4: inter-alpha-trypsin inhibitor heavy chain 4.**

### Analytical validation for the assays

The kits that showed detectable values were analytically validated. In those kits, the two pools described above were analysed 5 times at the same analytical run and 5 times on different days in order to evaluate the degree of imprecision intra-assay and inter-assay, respectively, assessed by the coefficient of variation (CV%) following the formula (CV% = standard deviation/mean*100).

The pools were also serially diluted to assess the accuracy of the assay by linearity under dilution. Results were analysed using a linear regression, and the linearity was evaluated by coefficient of determination (R^^2^^).

### Specific identification of ITIH4 fragments in porcine plasma and saliva by western blotting (WB)

#### Antibodies used in WB.

Two antibodies were used for the WB. (1) A commercial polyclonal antibody anti-human ITIH4 (Cloud-Clone Corp, Ref: PAH776Hu01, 0.45 mg/mL) with cross reactivity with porcine ITIH4 according to the manufacturer. This antibody was produced in rabbit using as an immunogen a recombinant ITIH4 (Glu29-Tyr157) expressed in *Escherichia coli.* (2) An in-house polyclonal antibody anti-porcine ITIH4 produced in sheep using the native swine ITIH4 purified at our laboratory as described below.

#### Purification of native ITIH4 from porcine plasma.

The purification of the native porcine protein was performed by an automated liquid chromatography system (AKTA pure, GE Healthcare Life Sciences) using an affinity column coated with the commercial recombinant polyclonal antibody. The column (HiTrap NHS-activated HP 1 ml, cytiva.com/hitrap, GE Healthcare Life Sciences, Munich, Germany) was coated following the manufacturer’s instructions and the native protein was subsequently purified from porcine plasma. Prior to passing through the column, the plasma was pooled and precipitated using ammonium sulphate (9.75g per 25mL of plasma) under agitation for 30 minutes. Then, it was centrifuged (3000g, 30 min, 25°C) and the supernatant was decanted. The precipitate was reconstituted in phosphate buffer saline (PBS) pH 7.4 and the buffer was exchanged to equilibrium buffer 20mM phosphate buffer pH 7.0 (NAP-25 DNA Columns, Cytiva, REF: 17-0852-02) and passed through the column. Subsequently, the purified protein was eluted by using a 0.1M glycine buffer (pH 2.5). The different fractions collected were then neutralised using neutralisation buffer (1M tris-base pH 9.0, Sigma-Aldrich) and concentrated with ultrafiltration centrifuge devices (Amicon® Ultra Centrifugal Filter, 50 kDa MWCO, UFC505008). The concentrated eluate was then buffer-exchanged to PBS (NAP-5 DNA Columns, Cytiva, REF: 17-0853-02).

The amount purified protein was 0.93 mg/mL, and it was quantified using the Bradford method in a standard plate reader (BioTek EPOCH2, Bio Tek Instruments, Inc., 100 Tigan Street, Microplate Spectrophotometer; Ref: EPOCH2NS).

#### Immunization and antibody production.

To produce the in-house polyclonal antibody in sheep a protocol previously described was followed [25]. A young female sheep around 9 months of age was selected to be immunized with the purified native ITIH4 protein mixed with incomplete Freund’s adjuvant in 1:1 proportion (Freund’s adjuvant, Incomplete; Sigma-Aldrich; Ref: F5506). The sheep was immunized 3 times (2 weeks between each inoculation), and plasma sample was collected from the jugular vein of the sheep. Plasma of sheep was tested in a common ELISA screening in order to see if the immunization was adequate and to evaluate the antibody titration. The amount injected was around 0.1 mg in a total maximum volume of 0.5 mL, administrated subcutaneously in different locations in order to avoid reactions. A HiTrap protein G HP affinity column was used to purify the polyclonal antibody from plasma of sheep obtained after blood extractions. PBS pH 7.4 was used as equilibrium buffer and citric acid 20mM as elution buffer according to the manufacturer’s instructions (GE Healthcare Life Sciences, Munich, Germany). Prior to passing through the column, the plasma of sheep was pooled and precipitated using ammonium sulphate (9.75g per 25mL of plasma) under agitation for 30 minutes. It was then centrifuged (3000g, 30 min, 25°C) and the supernatant was decanted. The precipitate was reconstituted in PBS and the buffer was exchanged to equilibrium buffer PBS pH 7.4 (NAP-25 DNA Columns, Cytiva, REF: 17-0852-02) and passed through the column. The different fractions collected were then neutralised using neutralisation buffer (1M tris-base pH 9.0, Sigma-Aldrich) and concentrated with ultrafiltration centrifuge devices (Amicon® Ultra Centrifugal Filter, 50 kDa MWCO, UFC505008). The concentrated eluate was then buffer-exchanged to PBS (NAP-5 DNA Columns, Cytiva, REF: 17-0853-02).

The amount of purified antibody was quantified using the Bradford method in a standard plate reader (BioTek EPOCH2, Bio Tek Instruments, Inc., 100 Tigan Street, Microplate Spectrophotometer; Ref: EPOCH2NS) and was 14.9 mg/mL.

### SDS-PAGE and western blotting procedures

Native ITIH4 purified in-house, plasma, and saliva from pigs were separated through three SDS-PAGE 12% acrylamide pre-cast gels (SurePAGE™, Bis-Tris, 10x8, 12%, 12 wells; Ref: M00668, Genescript, NJ, USA) and commercial running buffer (TRIS-MOPS-SDS Running buffer powder; Ref: M00138). Molecular weight marker used was a commercial protein standard marker (Amersham™ ECL™ Rainbow™ Marker-Full range, Cytiva, Ref: GERPN800E). Samples and protein were mixed with glycerol-70% and diluted with sample buffer (2% Sodium dodecyl sulfate (SDS), 1% blue bromofenol, 25% glycerol-70% and TRIS buffer 62.5mM pH 6.8) 1:1:1 proportion.

One of the gels was employed for protein identification distribution by Coomassie-stained with Coomassie Brilliant Blue R-250. Purified porcine ITIH4 for the stained gel was also denaturalized with 4% of dithiothreitol (GE Healthcare Life Sciences PlusOne DDT; Ref: 17-1318-02) heated 95º during 5 and 20 minutes.

The other two gels were transferred to two membranes by electroblotting using polyvinylidene difluoride membranes (PB9220, Power Blotter Pre-cut Membranes and Filters, PVDF mini, 0.45 μm pore, Invitrogen™). The membranes were overnight blocked with protein-free blocking solution (ROTI®Block, 250 mL 10x conc., ready-to-use). Then, one of the membranes was incubated with the commercial anti-ITIH4 antibody at 0.23 µg/mL; and the other membrane was incubated with the in-house polyclonal antibody at 0.25 µg/mL. Both antibodies were previously labelled with HRP using a commercial kit (Abcam, HRP Conjugation Kit – Lightning-Link®; Ref: ab102890) according to the manufacturer’s instructions (Abcam Limited, Campus Cambridge, UK).

The immunocomplexes formed were revealed with fresh solutions: A solution (luminol, 50 ml) and B solution (peroxide solution, 50 ml) (Western blotting Cytiva Lifescience™ Amersham™ ECL™). Stained gel and membranes were scanned using a camera imaging system for chemiluminescence western blotting and colorimetric image capture (Automatic molecular imaging system ImageQuant™ 500). Native ITIH4 protein purified in-house was used as positive control for the WB performed. Purified porcine Hp and calprotectin were used as negative controls, being analysed in the same conditions of the WB and they did not give any band that could be detected (data not shown).

### Statistical analysis

Descriptive parameters (mean, SD, CV or median and range), linear regression and R^^2^^ were calculated using routine statistical procedures. The distribution was assessed by the Shapiro–Wilk test and D’Agostino & Pearson normality test, showing a *p*-value <0.05. Therefore, the data were considered non-normal distributed and a non-*p*arametric approach was followed to analyze all the results. For comparison and assessment of the differences in salivary ITIH4 levels between the TB group and C group, the Mann–Whitney test was used. Results were expressed as median and range.

A correlation study between ITIH4 and Hp was performed using Spearman’s test for non-parametric data but only with the results of the kit which showed statistically significant differences in the comparison between groups (kit D). The degree of correlation was evaluated using the Rule of Thumb [26], which categorizes an R-value of 0.90 to 1 as indicative of a very high correlation, 0.70 to 0.90 as a high correlation, 0.50 to 0.70 as a moderate correlation, 0.30 to 0.50 as a low correlation and less than 0.30 a little if any correlation.

Data analyses for analytical validation were performed using spreadsheet software (Excel Version 10, Microsoft Corporation, Redmond, Washington, USA) and a statistics package (GraphPad Prism 6, GraphPad Software Inc., La Jolla, CA, USA).

## Results

### Analytical validation of the ELISA kits

All kits showed values higher than their limits of detection for saliva samples with the exception of kit B. Only the ELISA kits C and D were analytically validated since kit A did not show linearity in the measurements made in serial dilutions of saliva samples.

**Fig 1:**
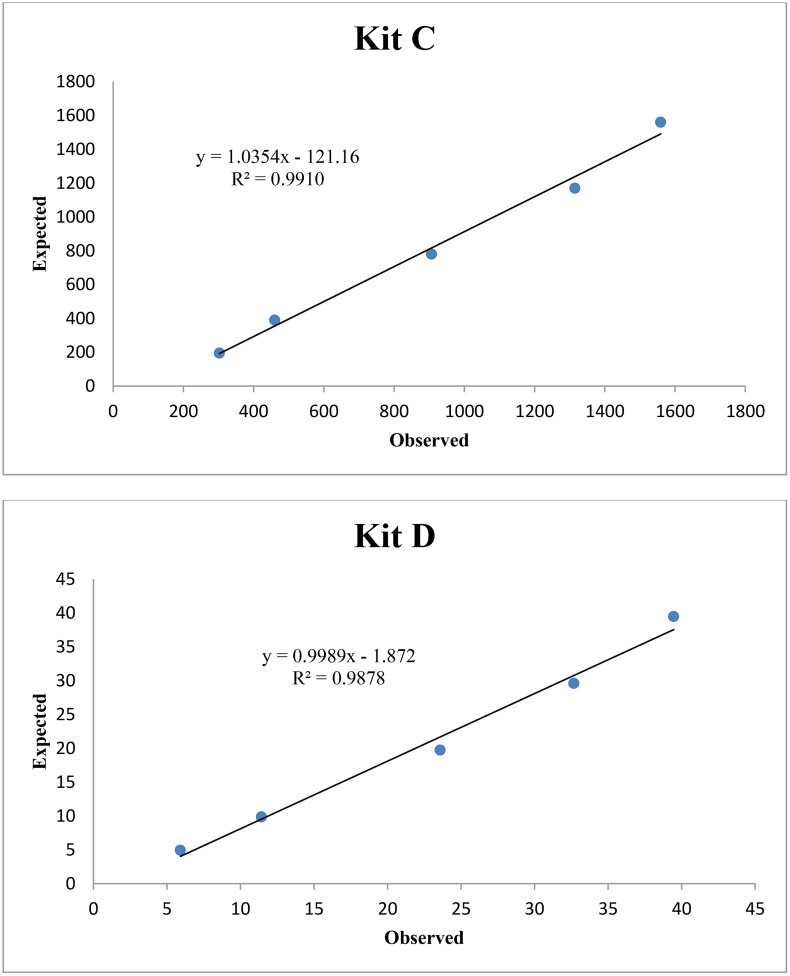
Serial dilutions of a porcine saliva pool sample with high ITIH4 concentration using the commercial ELISA kits C and D. On the x-axis are the observed values and on y-axis are the expected values. Units are in ng/mL.

The mean intra- and inter-assay imprecision of assays C and D are presented in Table 2. Both kits showed CV (%) below 14% in all cases and were able to detect the expected values of ITIH4 after serial dilutions of a saliva sample, showing determination coefficient close to 1 (Fig 1).

**Table 2:** Intra- and inter-assay imprecision of ITIH4 concentrations (ng/mL) measured by ELISA assays C and D in two porcine saliva pools with different concentrations of the analyte: high (PH) and low (PL).

		Intra-assay			Inter-assay		
ELISA	Sample	Mean	SD	CV (%)	Mean	SD	CV (%)
C	PH	1131.15	79.19	7.00	930.10	83.17	8.94
	PL	427.08	34.61	8.10	469.70	28.60	6.09
D	PH	62.42	8.31	13.32	81.54	7.56	9.27
	PL	7.93	1.02	12.91	11.66	1.54	13.26

Samples were pre-diluted with a dilution factor of 50 and with a dilution factor of 4 for kit C and D, respectively. PH: pool high; PL: pool low; SD: standard deviation; CV: coefficient of variation.

**Fig 2:**
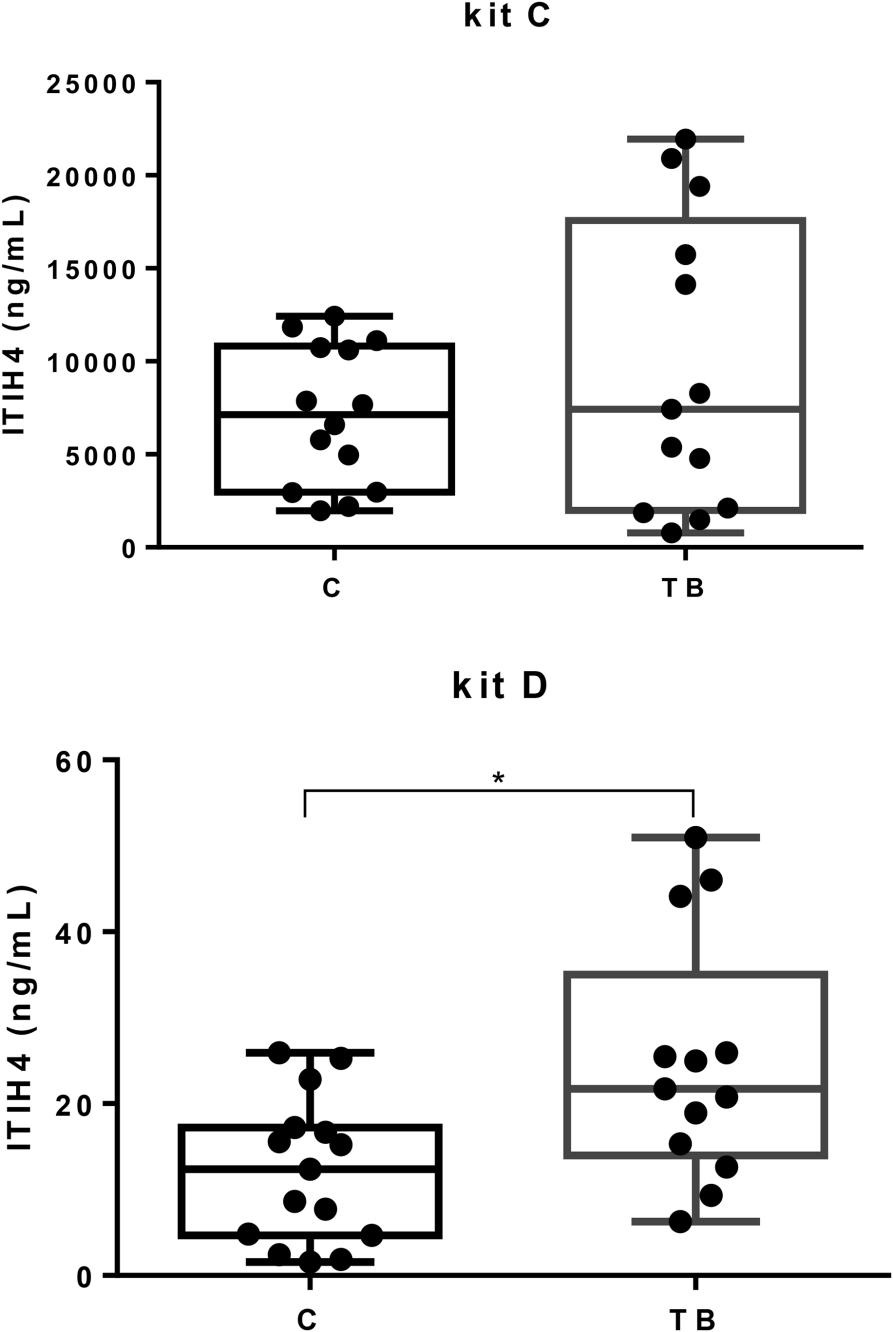
Salivary ITIH4 concentrations of pigs with tail biting lesions (TB) and healthy pigs (Control). Boxes show the interquartile range (25th and 75th percentile), the line within the boxes the median values, and the whiskers indicate the range (minimum and maximum value). Saliva samples were pre-diluted with a dilution factor of 50 for the measurements of kit C and 4 for the measurements of kit D, and the raw result obtained from the kits was multiplied by dilution factor in order to give the real value of the saliva samples.

For kit D, pigs with tail biting showed higher levels of ITIH4 than the control group (*p* < 0.05). However, no statistically significant differences were shown between the two groups when kit C was used (Fig 2). A moderate correlation between ITIH4 values measured with kit D and Hp was found (Table 3).

**Table 3:** Correlation data (correlation coefficient and correlation *p*-value) between salivary ITIH4 concentrations measured with the kit D (ng/mL) and salivary Hp (ng/mL) in pigs with tail biting (TB) and healthy pigs (C).

Groups	Biomarker	Mean	Range	Spearman (r)
TB	Hp	8108.15	(6246-10410)	0.58 (*p *= 0.001)
	ITIH4	24.81	(6.28-50.99)	
C	Hp	1879.69	(728-3912)	
	ITIH4	12.19	(1.58-2593)	

### ITIH4 distribution in saliva

**Fig 3:**
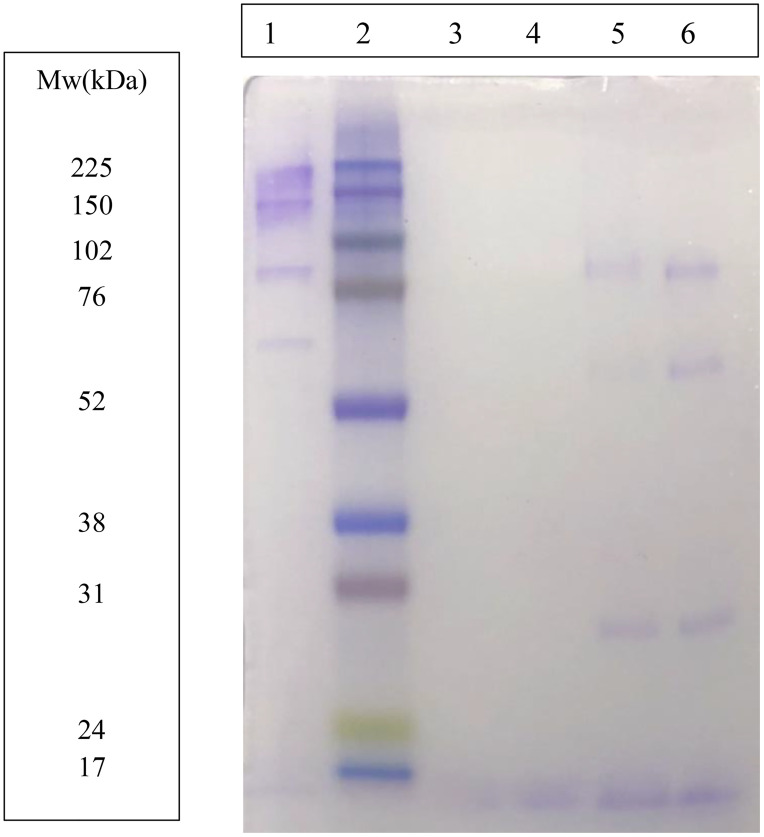
SDS-PAGE image of the purified porcine ITIH4. Lane 1, the purified protein; lane 2, marker; lane 3 and 4 the purified protein pre-diluted 1/10 and 1/100, respectively; lane 5 and 6 the purified protein with DDT heated 5 and 20 minutes, respectively. The image is not readjusted.

The purified protein showed four main bands using SDS: (1) around 200 kDa; (2) around 120 kDa; (3) around 80 kDa; and (4) around 60 kDa. After denaturalization using DTT and heated at 95ºC, bands 3 and 4 were maintained, and an additional band was seen (5) around 30 kDa (Fig 3).

**Fig 4:**
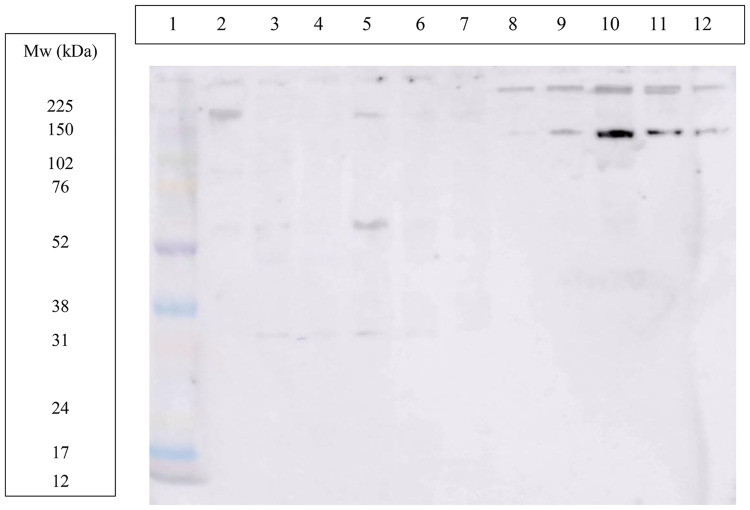
Western blot using a commercial polyclonal antibody produced against recombinant ITIH4. Lane 1: molecular weight marker; lane 2: in-house purified porcine ITIH4 0.93 mg/mL; lane 3, 4, 5, 6 and 7: porcine saliva samples from different animals; lane 8, 9, 10, 11 and 12: porcine plasma samples from different animals.

**Fig 5:**
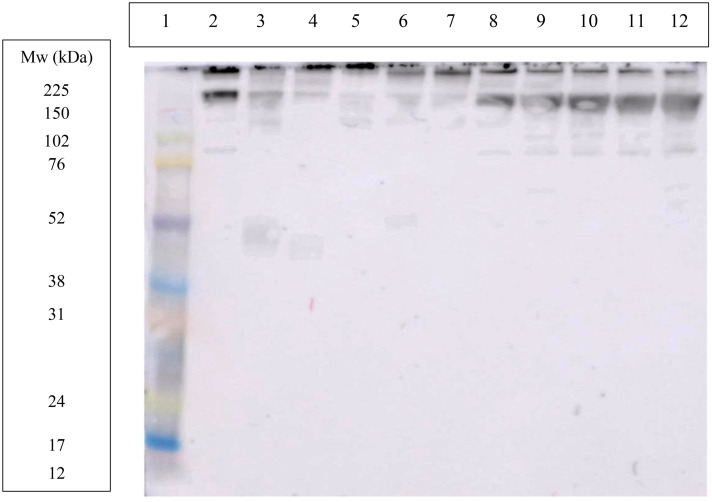
Western blot using the in-house sheep polyclonal antibody produced against purified porcine ITIH4. Lane 1: molecular weight marker; lane 2: in-house purified porcine ITIH4 0.93 mg/mL; lane 3, 4, 5, 6 and 7: porcine saliva samples from different animals; lane 8, 9, 10, 11 and 12: porcine plasma samples from different animals.

In WB, both antibodies, the commercial (Fig 4) and the antibody developed in our report (Fig 5) showed in the lane corresponding to purified ITIH4 one band around 200 kDa and other around 80 kDa. However, the antibody developed in our report showed a higher intensity of the bands and also a band around 120 KDa that did not appear with the commercial antibody.

When the commercial antibody was used, bands around 200 and 120 kDa were detected in lanes corresponding to plasma samples (lanes 8–12, Fig 4). The antibody developed in this study produced the same two bands as the commercial antibody, as well as bands around 80 and 60 kDa (lanes 9 and 12, Fig 5).

When the commercial antibody was used, bands at 200, 60 and 30 kDa were detected in lanes corresponding to saliva samples (lanes 3–7, Fig 4). Using the antibody developed in our laboratory, bands were observed at around 200 and 120 kDa, as well as between 60 and 30 kDa in some samples (lanes 3, 4 and 6, Fig 5).

## Discussion

In this work, four different ELISA assays for ITIH4 quantification were tested in saliva samples. Two kits produced a detectable signal in saliva and quantified ITIH4 with adequate precision (CVs were under 20% of imprecision intra- and inter-assay) and had a coefficient of determinations of linear regression equations close to 1, indicating an adequate accuracy [27]. The reason why some kits were not able to quantify ITIH4 in saliva samples may be related to the use of different antibodies and assay formats that may have different affinity for the protein, and in some cases may not be sufficient to quantify the amounts of this protein in saliva, or even the conformation of the protein in this type of sample makes the antibodies unable to detect it adequately. The two commercial ELISA assays that gave adequate precision and accuracy in the analytical validation, showed higher concentrations of ITIH4 in animals that suffered from tail biting compared to controls. However, differences were only significant using the kit D. This kit D has been previously demonstrated to show increases in ITIH4 in porcine saliva in pigs with diarrhoea due to *E.coli* [17], and meningitis by *S.suis* infection [18] compared to healthy animals, and also under stress situations such as after 4 hours of lairage in the slaughterhouse regarding to basal levels [19]. These results support the ability of this kit to quantify and detect increases of ITIH4 in saliva of pigs.

Our report revealed a moderately significant correlation in saliva between the ITIH4 measured with kit D and Hp, a known APP in pigs [23]. A correlation between ITIH4 and Hp has also been previously reported in serum of pigs [14]. This would support the idea that ITIH4 in saliva is involved in inflammation as an APP as occurs in serum. In the inflammatory process, ITIH4 is produced by interleukins [28,29] and has various functions such as anti-apoptotic, matrix stabilizing, repairing tissue and liver formation [9,30]. The increases found in ITIH4 in the pigs with tail biting were of around 2-fold compared to the control group, that would be in line with the magnitude of other previous increases reported in the saliva of pigs with *E. coli* [17] and *S. suis* infection [18] as well as in tail biting [20] and they are lower than the 3–4 fold increased previously described in a report about *E. coli* lipopolysaccharide (LPS) administration [19]. Overall, this data would support the moderate increases of this APP in inflammation.

In this report, we produced a polyclonal antibody that was species-specific against porcine ITIH4 purified from plasma. This allowed us to have an additional and more specific tool to evaluate the different ITIH4 forms than the commercially available antibody, that was raised against a recombinant protein made with the sequence of human ITIH4, and that was also used in our study for comparative purposes. This polyclonal antibody was produced in sheep, which is a species previously used for the development of antibodies against other APPs [31] and that among other advantages it allows to obtain larger amounts of antibodies.

Compared to the commercial antibody, the polyclonal antibody produced in our report showed a higher intensity of the bands (probably to its high affinity to the porcine ITIH4) and also different patterns of bands in each different sample types tested (purified protein, plasma and saliva). However, all the bands showed across the different sample types (200, 120, 80, 60 and between 60 and 30 kDa) could reflect the different components described for this protein.

- The band that appeared at around 200 kDa might be due to the fact that ITIH4 could form different complexes with other proteins such as proteases [28,29] and therefore appear at higher molecular weights. In this line, it has been reported that ITIH4 can form complexes with kallikrein [28].
- The band at 120 kDa has been reported previously in humans and it is homologous to the ITIH4 found in porcine species [1].
- The 80 kDa band would correspond to the 80–85 kDa band described previously as a fragment of ITIH4 in humans [5] and pigs [6], showing an identical sequence to the 120 kDa human ITIH4 [6].
- The band around 60 kDa, was similar to a band of ITIH4 previously described [1] and identified as different reactant peptides produced from the protein of 120 kDa [2,6].
- Regarding the bands between 30 kDa and 60kDa, they could correspond to fragments of ITIH4 produced in porcine plasma after digestion with plasma kallikrein [10].

Overall, the presence of bands of lower molecular weight than 120 kDa would due to fragments of ITIH4 that are cleaved by different proteases as it has been demonstrated in humans [29].

The differences in the patterns of the bands showed by each antibody in the variety of sample types could be due to the distinct nature of the antibody. In this line the antibody produced in our laboratory revealed bands of 80 and 60 kDa in plasma and the band of 120 kDa in saliva that did not appear when the commercial antibody was used. These differences could be due to the fact that the antibody produced in our laboratory recognizes fragments of the protein that are not present in the human sequence, or that differ slightly in sequence or conformation, since the in-house antibody has been produced specifically against the native porcine protein, unlike the commercial one.

In addition, there was a different distribution of ITIH4 forms depending on the sample type with saliva samples showing additional bands of lower molecular weight than 60 kDa that did not appear in plasma. The presence of these bands raises questions about the potential roles of these fragments in pig saliva and their relevance in the biological context. Thus, additional future research is necessary to clarify the reasons for their presence, their functions and roles in both fluids. In addition to the different band distribution between saliva and plasma, the concentration of ITIH4 differs between both fluids in pigs, with values in healthy animals in serum of 1–3 g/L [32] which are higher than the values in saliva found in our report 1.58–25.93 ug/L. This is in agreement with the higher values in serum compared to saliva that have been described in other APPs such as CRP or Hp in swine [16].

This report has various limitations. One is that the results are valid for the batches of the commercial kits used in our conditions, and other different batches or future presentations of the kits could provide different results. Also, the results of WB were no confirmed in mass spectrometry in order to assess if the bands had ITIH4 and therefore, unspecific staining of other proteins or protein subunits could occur. Further research involving a larger number of pigs with different inflammatory conditions should be made to clarify the possible use of the measurement of ITIH4 in the saliva of pigs as a biomarker to detect and monitor inflammation.

## Conclusion

This report provides evidence of the presence of ITIH4, also known as Pig-Map, in saliva of pigs indicating that although it has some components similar to serum, it also contains different low molecular weight fragments, whose roles in saliva would be of interest to be clarified in the future. In addition, it shows that different antibodies can have different reactivities for this protein and also for its different components, which could be one of the reasons for the different performance of the kits used in this report to detect ITIH4. Finally, this study indicates that there is a commercial kit that can measure ITIH4 in saliva with adequate precision and accuracy, and also it can detect increases in this protein in situations of tail biting. These data confirm that ITIH4 can be detected in saliva and be potentially used as a marker of inflammation.

## Supporting information

S1 File Raw images. (PPTX)

## Abbreviations

APPs: Acute phase proteins
CRP: C-reactive protein
R^2^: Coefficient of determination
CV: Coefficient of variation;
*E.coli*: *Escherichia coli*
sgp120: Glycoprotein 120 KDa
Hp: Haptoglobin
HRP: Horseradish peroxidase
ITIH4: Inter-alpha-trypsin inhibitor protein heavy chain 4
IHRP: ITI heavy chain human-related protein
LPS: Lipopolysaccharide
SAA: Serum amyloid A
PBS: Phosphate buffer saline
MAP: Porcine major acute phase protein pig
PRRSv: Porcine reproductive and respiratory syndrome virus
SDS: Sodium dodecyl sulfate
SD: Standard deviation
*S.suis*: Streptococcus suis
WB: Western Blotting/ Western Blot
