# Supplementary material for: Inter-alpha-trypsin inhibitor heavy chain 4 (ITIH4) in saliva of pigs: evaluation of two commercially available ELISA kits for its measurement and distribution of its main components
Source: PLoS One. 2025 Oct 24;20(10):e0335133. doi: 10.1371/journal.pone.0335133 (PMC12551830; doi:10.1371/journal.pone.0335133)
Supplement: S1 File — (PPTX) [file pone.0335133.s001.pptx]

## Slide 1
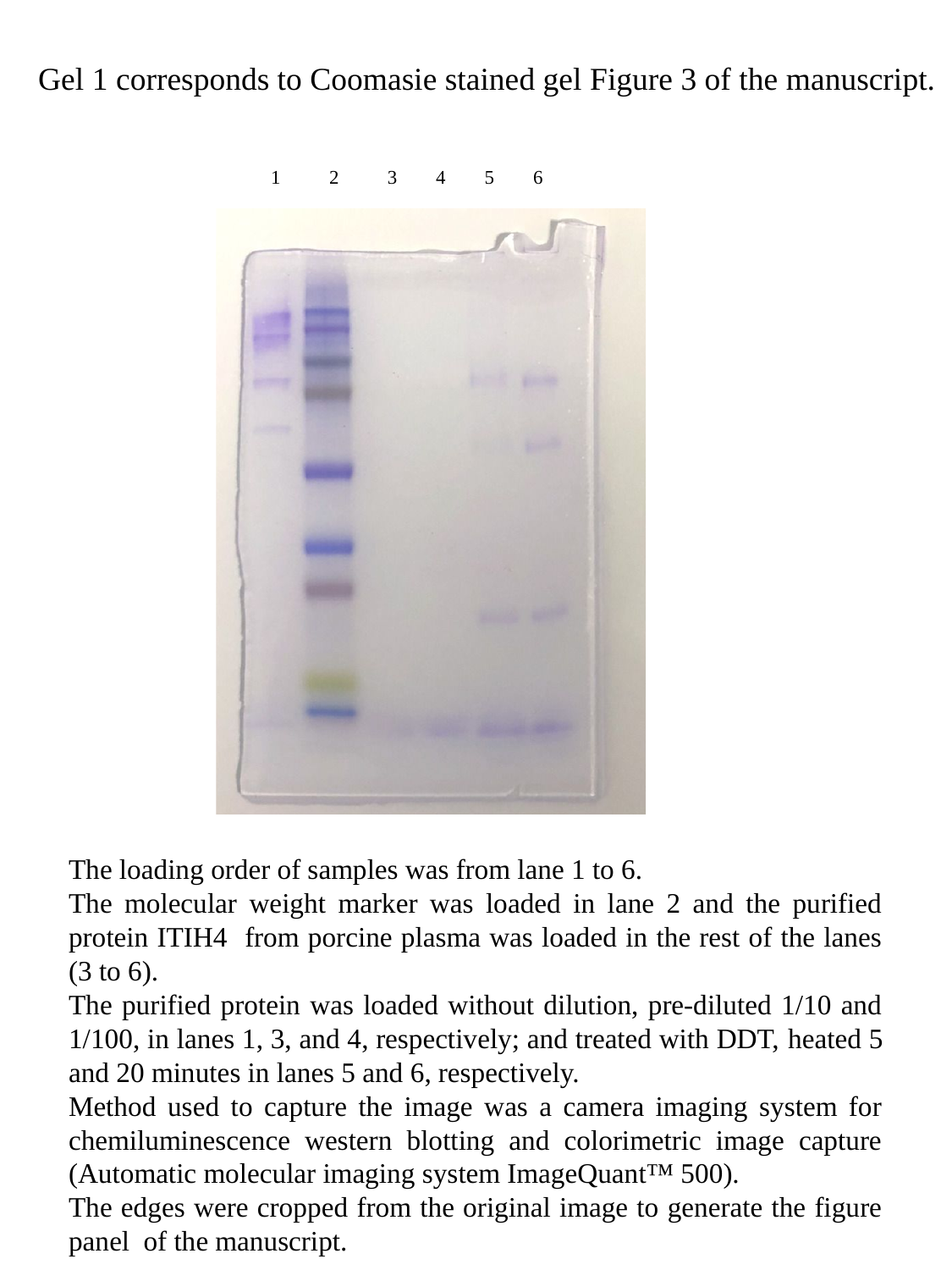

Gel 1 corresponds to Coomasie stained gel Figure 3 of the manuscript.
1 2 3 4 5 6
The loading order of samples was from lane 1 to 6.
The molecular weight marker was loaded in lane 2 and the purified protein ITIH4 from porcine plasma was loaded in the rest of the lanes (3 to 6).
The purified protein was loaded without dilution, pre-diluted 1/10 and 1/100, in lanes 1, 3, and 4, respectively; and treated with DDT, heated 5 and 20 minutes in lanes 5 and 6, respectively.
Method used to capture the image was a camera imaging system for chemiluminescence western blotting and colorimetric image capture (Automatic molecular imaging system ImageQuant™ 500).
The edges were cropped from the original image to generate the figure panel of the manuscript.

## Slide 2
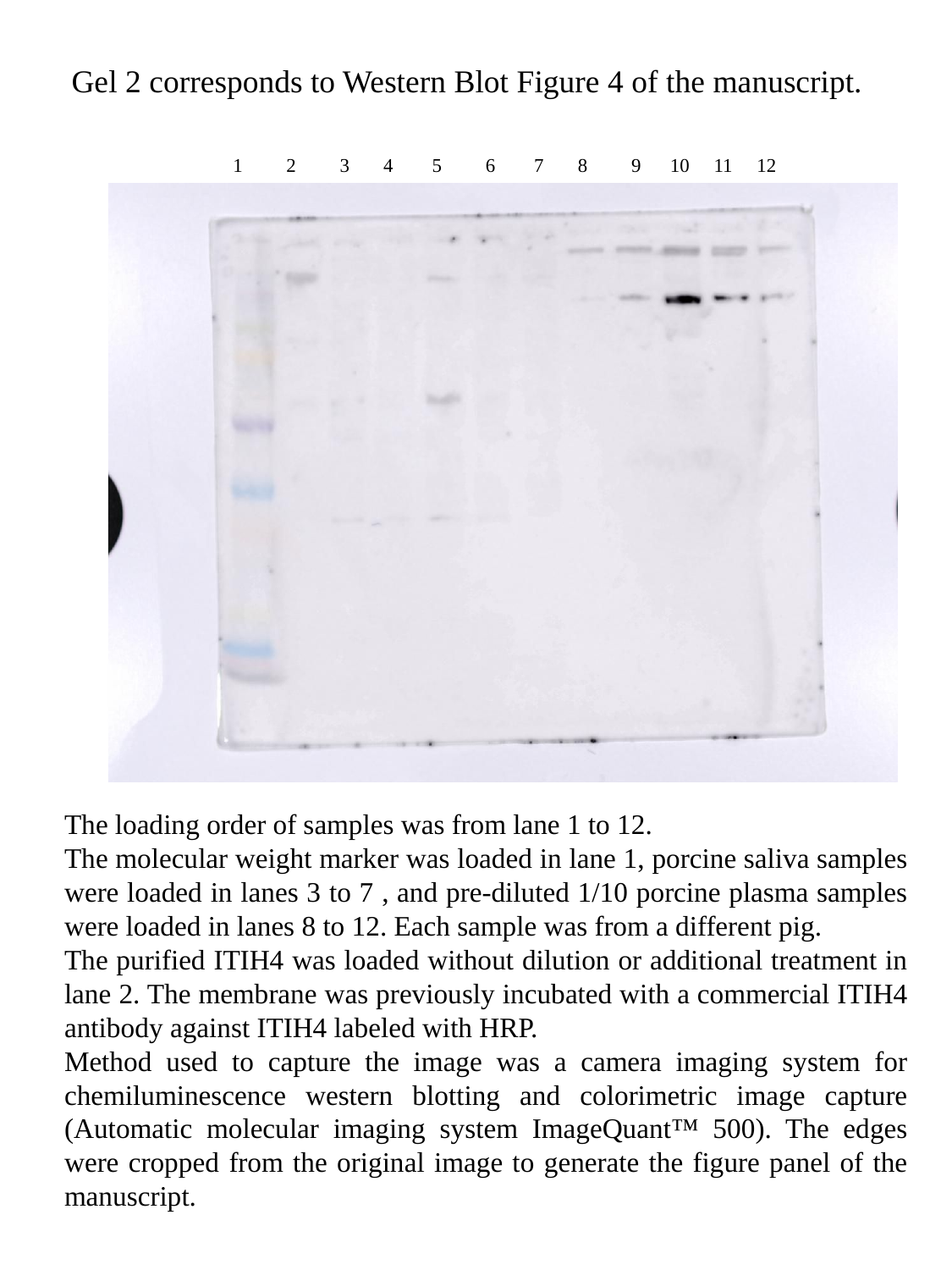

Gel 2 corresponds to Western Blot Figure 4 of the manuscript.
 1 2 3 4 5 6 7 8 9 10 11 12
The loading order of samples was from lane 1 to 12.
The molecular weight marker was loaded in lane 1, porcine saliva samples were loaded in lanes 3 to 7 , and pre-diluted 1/10 porcine plasma samples were loaded in lanes 8 to 12. Each sample was from a different pig.
The purified ITIH4 was loaded without dilution or additional treatment in lane 2. The membrane was previously incubated with a commercial ITIH4 antibody against ITIH4 labeled with HRP.
Method used to capture the image was a camera imaging system for chemiluminescence western blotting and colorimetric image capture (Automatic molecular imaging system ImageQuant™ 500). The edges were cropped from the original image to generate the figure panel of the manuscript.

## Slide 3
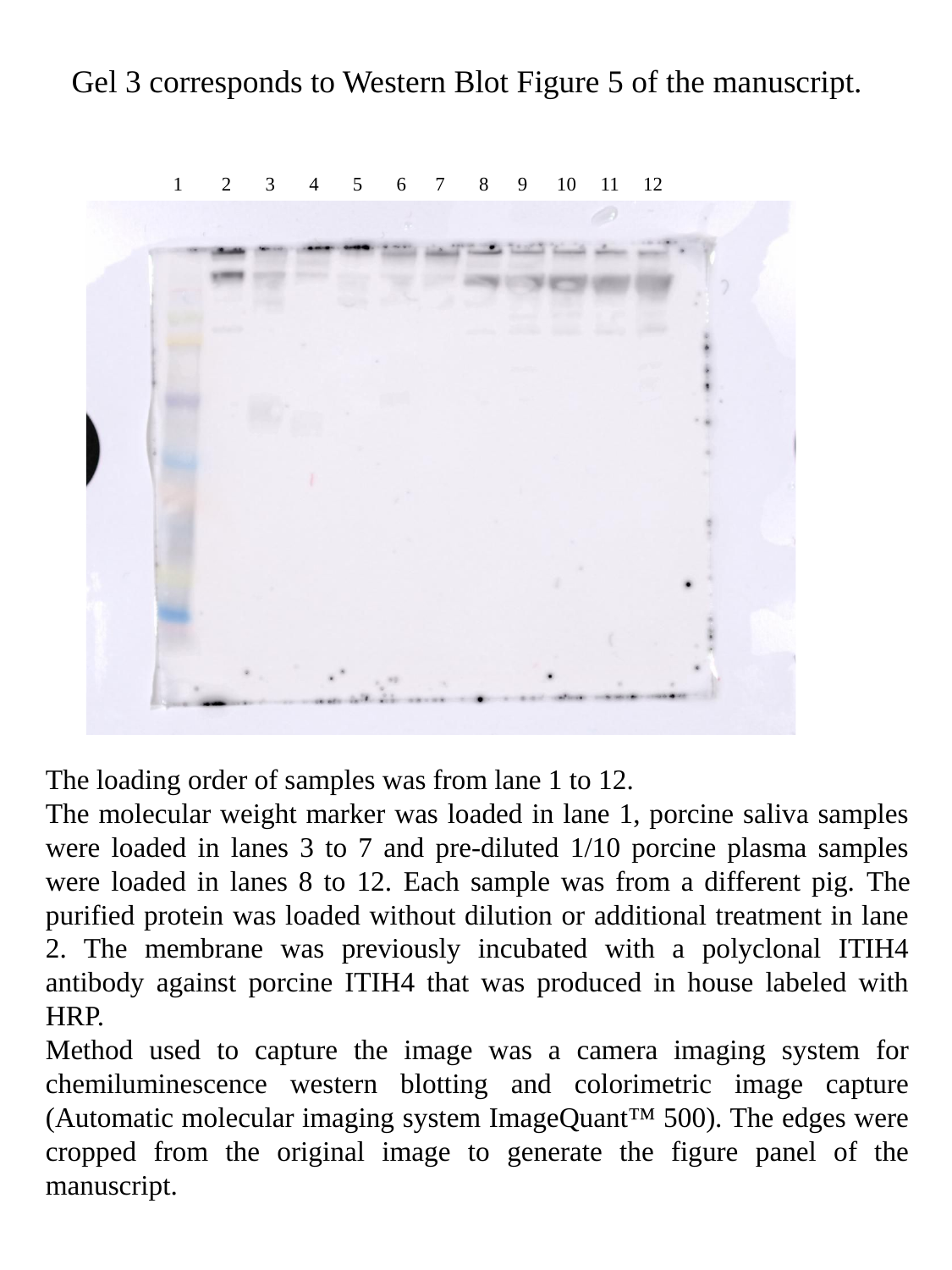

Gel 3 corresponds to Western Blot Figure 5 of the manuscript.
 1 2 3 4 5 6 7 8 9 10 11 12
The loading order of samples was from lane 1 to 12.
The molecular weight marker was loaded in lane 1, porcine saliva samples were loaded in lanes 3 to 7 and pre-diluted 1/10 porcine plasma samples were loaded in lanes 8 to 12. Each sample was from a different pig. The purified protein was loaded without dilution or additional treatment in lane 2. The membrane was previously incubated with a polyclonal ITIH4 antibody against porcine ITIH4 that was produced in house labeled with HRP.
Method used to capture the image was a camera imaging system for chemiluminescence western blotting and colorimetric image capture (Automatic molecular imaging system ImageQuant™ 500). The edges were cropped from the original image to generate the figure panel of the manuscript.
